# Screening of Bioactive Fraction of Radix Paeoniae Alba and Enhancing Anti-Allergic Asthma by Stir-Frying Through Regulating PI3K/AKT Signaling Pathway

**DOI:** 10.3389/fphar.2022.863403

**Published:** 2022-03-31

**Authors:** Xia’nan Sang, Jialiang Ying, Xuedong Wan, Xin Han, Qiyuan Shan, Qiang Lyu, Qiao Yang, Kuilong Wang, Min Hao, Erlong Liu, Gang Cao

**Affiliations:** School of Pharmacy, Zhejiang Chinese Medical University, Hangzhou, China

**Keywords:** Radix Paeoniae Alba, stir frying Radix Paeoniae Alba, PI3K, AKT, allergic asthma

## Abstract

Allergic asthma is a common respiratory inflammation disease. The crude Radix Paeoniae Alba (RPA) and its processed products have been used frequently as antipyretic and anti-inflammatory agents in traditional medicine. To evaluate the effect of honey and bran processing, different fractions of RPA were used for treating anti-allergic asthma in the ovalbumin (OVA)-induced mice model, and then, the most effective fraction of RPA and stir-frying Radix Paeoniae Alba with honey and bran (FRPA) for treating anti-allergic asthma were compared mutually for pharmacological effects. The results showed that the treatment of the dichloromethane fraction of RPA significantly improved the pathological condition of lung tissues, decreased the number of eosinophils and other cells in bronchoalveolar lavage fluid (BALF), and the increased the expression of various inflammatory factors. Furthermore, the study discovered that the lung pathological conditions, compared with the high dose of dichloromethane RPA fraction, could be ameliorated by high dose of dichloromethane FRPA fraction treatment. Moreover, the expression of inflammatory factors and the phosphorylation of the PI3K/AKT signaling pathway could be diminished by FRPA. Finally, the contents of compounds with a significant difference in the FRPA dichloromethane fraction were paeoniflorin, ethyl gallate, pentagalloylglucose, galloylpaeoniflorin, and others by UPLC/Q-TOF-MS analysis. These findings suggest that the dichloromethane fraction of FRPA has an enhancement effect on anti-allergic asthma and provide the experimental basis for exploring the processed mechanism of RPA.

## 1 Introduction

Radix Paeoniae Alba (RPA), the dried root of *Paeonia lactiflora* Pall, is a well-known natural medicine for more than 1,200 years in China. Processing of RPA is a pharmaceutical technique that transforms medicinal raw materials into decoction pieces for use in clinical application. There are many processing methods of RPA, including wine-made RPA, FRPA, stir-frying RPA, and wine–bran-made RPA. In the long-term clinical use, FRPA can relieve pain, strengthen the spleen, and soften the liver. Over the past few decades, there has been a rapid growth in the information available on the pharmacological activities of RPA. Studies have shown that RPA displays a wide spectrum of pharmacological effects, including anti-inflammation, pain easing, liver protection, and anti-oxidation (Ye et al., 2020; Gu et al., 2021). These pharmacological effects laid the foundation for RPA being a potential therapeutic agent for the treatment of several diseases, such as MPTP-induced experimental parkinsonism (Zhang et al., 2019), atopic dermatitis (Jo et al., 2018), asthma (Wang et al., 2021), depression (Zhang et al., 2020), and hepatic disorders (Wang et al., 2012).

Modern research indicates that monoterpenes, flavonoids, tannins, triterpenes, and polysaccharides are the main components of RPA related to the pharmacological activities of Paeoniae Radix (Ye et al., 2020; Gu et al., 2021). Monoterpene glycosides as the major characteristic compounds of RPA display a wide pharmacological effect, including anti-inflammatory and antitumor activities. Total glucosides of peony were isolated from RPA, which had been approved for treating rheumatoid arthritis (RA) by the State Food and Drug Administration of China. Galloylglucose is an important compound with significant biomedical benefits, such as being a free radical sink, an anti-inflammatory agent, anti-diabetic agent, and enzymatic resistant properties (Patnaik et al., 2019). Paeonol is one of the main polyphenolic compounds in RPA, and its injection has been successfully applied in China for nearly 50 years for inflammation/pain-related indications (Zhang et al., 2019). The extensive biological activities made RPA a therapeutic agent to be widely used in clinical therapy. Modern pharmacological studies show that RPA has been suggested to play an important role in the treatment of asthma (Wang et al., 2021).

Asthma is a common heterogenic disease caused by chronic inflammation in the lower respiratory tract. Its characteristics include variability of airway obstruction and high reactivity of the bronchus. Asthma often occurs in children younger than 10 years, and the prevalence rate is about 10%. Patients with asthma are characterized by repeated cough, wheezing, shortness of breath, chest tightness, etc., and lung cancer, which affects tens of thousands of people all over the world, has a great correlation with asthma (Mims, 2015; Qu et al., 2017). As one of the most common asthma types, allergic asthma has airway inflammation and changing choroidal structure manifestation (Yılmaz et al., 2021), whose sensitization mainly affects mast cells and histamine, trypsin, etc., which are treated by a variety of therapies, such as inhaled corticosteroids, budesonide, and fluticasone in the clinic. In addition, β2 receptor agonists, such as salbutamol, pirbuterol, and formoterol, and leukotriene regulators, such as half-skin acid leukotriene 1 receptor blockers and 5-lipoxygenase inhibitors, are widely used to treat allergic asthma (Hamid et al., 2003; Han and Wang, 2021).

RPA is already widely available for the management of respiratory conditions, mainly regarding inflammation and immune function symptoms. In this study, the research aimed to evaluate the effects of each fraction from RPA for anti-allergic asthma by using the OVA-sensitized mice model and further investigated whether the most active fraction from processed products possesses the enhancement of the pharmacological effects after stir-frying with honey and bran. At the same time, the inflammatory factors and signaling pathways of the processed products increased pharmacological actions, and the comparison about the contents of the main components in the active fraction from RPA and FRPA for anti-allergic asthma were studied.

## 2 Materials and Methods

### 2.1 Preparation of Radix Paeoniae Alba and Fried Radix Paeoniae Alba Extracts

**FIGURE 1:**
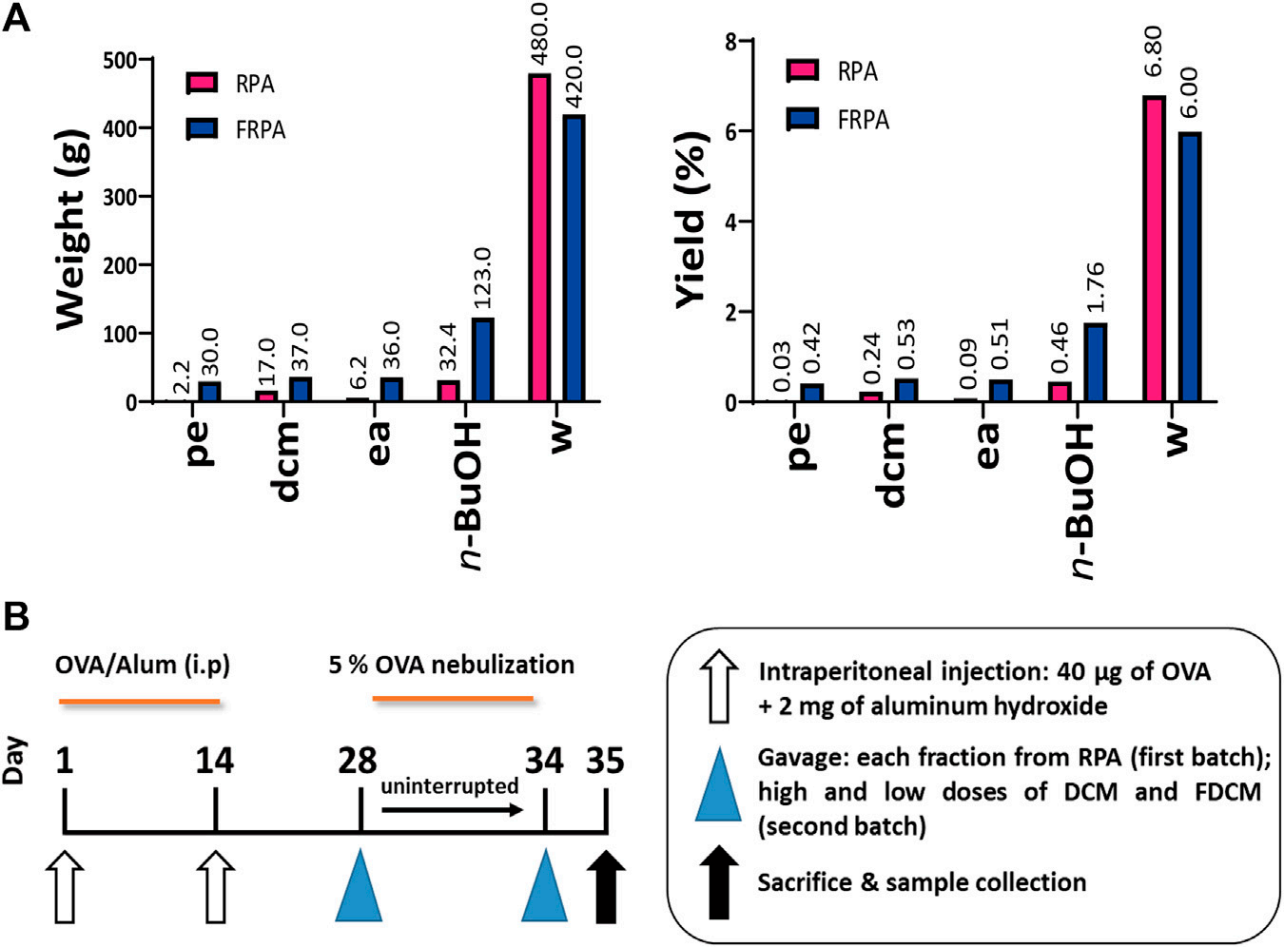
Weight, yield and outline of experimental design. **(A)** Weight and yield of RPA and FRPA fraction; the small letters of pe, dcm, ea, *n*-BuOH, and w are short forms for petroleum ether, dichloromethane, ethyl acetate, *n*-butanol, and water fraction, respectively. **(B)** Outline of experimental design for establishing the OVA-sensitized allergic asthma model, and an illustration of critical points during the experimental timeline.

RPA and FRPA were purchased from Zhejiang University of Chinese Medicine Chinese Herbal Pieces Co., Ltd. Hangzhou City, Zhejiang. The dried herbs (7 kg) were exhaustively extracted two times with 70% (v/v, 5 × 10 L) ethanol by refluxing for 2 h. The supernatant was collected and concentrated under reduced pressure, using a rotatory evaporator, yielding crude RPA and FRPA extract. The extract of RPA was then dispersed in water and successively partitioned with 4 L of petroleum ether fraction (PE), dichloromethane fraction (DCM), ethyl acetate fraction (EA), *n*-butanol fraction (*n*-BuOH), and water fraction (W), respectively. The extraction time of each extractant was controlled at 3 h. In addition, the abbreviation of FRPA for each fraction is as follows FPE, FDCM, FEA, F*n*-BuOH, and FW. Decompressing and concentrating the fractions of each fraction and the water fraction obtained the extract concretes of RPA and FRPA. The weight and yield of RPA and FRPA fractions are shown in Figure 1A.

### 2.2 Establishment and Drug Treatment of Allergic Asthma Model in Mice

All animal protocols involved in this experiment were approved by the Laboratory Animal Research Center of Zhejiang Chinese Medicine University, and the experiments were conducted in strict accordance with the “Guide for the Care and Use of Laboratory Animals” issued by the U.S. National Institute of Health.

Female BALB/c mice were purchased from SLAC Laboratory Animal Co., Ltd. (Shanghai, China). OVA was a common allergen, which was often used to establish an allergic asthma model (Chen et al., 2021). The overview of animal experiments is given in Figure 1B. The research established the OVA model for the first batch; sixty female mice, six to 8 weeks old and weighing about 22 g, were sensitized with an OVA prescription containing 0.2 mg/ml OVA in an aluminum hydroxide gel, and 0.2 ml suspension was intraperitoneally injected into the abdominal cavity at day 1 and day 14. For seven uninterrupted days from day 28 to day 34, the sixty sensitized female mice were divided into six groups, with ten mice per group: OVA-sensitized model, PE treatment (6.84 mg/kg), DCM treatment (54.72 mg/kg), EA treatment (20.06 mg/kg), *n*-BuOH treatment (104.88 mg/kg), and W treatment (1,550.40 mg/kg). Each group, except the OVA-sensitized model group, received the treatment, respectively, which was administered by using the oral gavage method with each fraction from RPA. Then, sixty mice were allowed to inhale 5% atomized OVA for 30 min in an exposure cage. In addition, another ten non-sensitized female BALB/c mice were assigned to the normal control group. After 7 days, the relevant index was observed.

The research established the OVA model for the second batch to further study; sixty female mice were used in this batch, and fifty sensitized female mice were divided into five groups, with ten mice per group: OVA-sensitized model, high DCM dose (HD, 57.33 mg/kg), low DCM dose (LD, 28.67 mg/kg), high FDCM dose (HFD, 127.34 mg/kg), and low FDCM dose (LFD, 63.67 mg/kg). The remaining methods of the establishment of the OVA model were the same as the first batch.

### 2.3 Collection and Analysis of Mouse Bronchoalveolar Lavage Fluid and Blood

The mice’s blood was taken from the heart after anesthesia and then the blood was centrifuged at 3,000 rpm for 10 min to gain serum samples. After blood collection, the mice were dissected, the mice lung was accurately found, and the right lung was ligated. The left lung was lavaged three times with 0.4 ml phosphate-buffered saline (PBS). Then 1% glacial acetic acid was used to dilute the aliquot of the lavage fluid samples, and the microscope with cell counting slides was used to determine the total leukocyte numbers. To research further, the BALF samples were loaded onto the slides and dried to stain with the Wright–Giemsa stain. Finally, the optical microscope was used to count the number of total monocytes, eosinophils, and neutrophils at high magnification.

### 2.4 Serum Cytokine Assay by Enzyme-Linked Immunosorbent Assay

According to the manufacturer’s instructions, the quantification analysis included IL-17, IL-4, and IgE, which were monitored using the mouse enzyme-linked immunosorbent assay kit (MEIMIAN). The determination results were substituted into the regression equation of the standard curve and finally multiplied by the dilution multiple to obtain the contents of each fraction.

### 2.5 Pathological Analysis of Lung Tissue in Mice

The mice lung was removed by dissection and fixed in 4% paraformaldehyde. Then paraffin was used to embed the lung tissues, and they were cut into sections. Next, the mice tissue sections were stained with hematoxylin and eosin (H&E) to analyze histopathology. After staining, the optical microscope was used to analyze the degree of inflammatory infiltration in the tissue sections.

### 2.6 Western Blotting Explored the Regulation of Inflammatory Factors and Signaling Pathway in Fried Radix Paeoniae Alba That Enhances Anti-Allergic Asthma

In this research, the lung tissues were lysed with RIPA Lysis Buffer and used protein extraction reagent to extract the total protein of tissues. Then, the homogenates were centrifuged at 12,000 rpm for 10 min at 4°C, the supernatants were collected, and a bicinchoninic acid (BCA) protein assay was used to measure the protein concentration. The proteins were separated by 12% sodium dodecyl sulfate–polyacrylamide gel electrophoresis (SDS-PAGE) and 5% concentrated glue, and the proteins were transferred onto polyvinylidene fluoride (PVDF) membranes at appropriate electric current and time. Then, the PVDF membranes were blocked in 5% skim milk for 2 h at about 20°C. After blocking, the membranes were washed with phosphate-buffered saline with Tween-20 (PBST) for 30 min and then incubated with appropriate primary antibodies overnight on the shaking bed of 4°C refrigerators. The primary antibodies used were as follows: IL-1β, IL-6, p-PI3K, PI3K, p-AKT, AKT, and GAPDH. The next day, the membranes were washed with PBST and incubated with a 1:10,000–1:5,000 dilution of appropriate secondary antibody for 1–2 h on a shaker at 20°C. After incubating, the membranes were washed with PBST three times, and then the enhanced chemiluminescence (ECL) was used to visualize the proteins of membranes under the Western blotting detection system. The protein bands were analyzed using ImageJ.

### 2.7 Immunofluorescence Analysis of Fried Radix Paeoniae Alba Lung Tissue Sections

The lung tissue was cut to a thickness of 5 µm by using a cryomicrotome. The sections were subsequently blocked with 5% goat serum for 30 min and incubated overnight with the p-AKT (1:500 dilution) antibody at 4°C. The next day, after washing three times with PBS, the sections were incubated with appropriate secondary antibodies (1:500 dilution) at room temperature for 2 h. Then, the sections were washed three times with PBS and finally mounted with UltraCruz mounting media containing DAPI. The confocal fluorescence microscope was used in this experiment.

### 2.8 The Changed Contents of Main Compounds of the Most Active Fraction of Anti-Allergic Asthma After Stir-Frying in Fried Radix Paeoniae Alba Compared With Radix Paeoniae Alba

The samples of the most active fraction of anti-allergic asthma in RPA and FRPA would be analyzed by UPLC/Q-TOF-MS to detect the liquid chromatograms of the characteristic peaks. Meanwhile, the components and contents of the compounds were identified by UPLC/MS software and the supports of the Pharmacopoeia standards and the compounds reported in the references. Finally, the research summarized some compounds with significantly different contents after stir-frying with honey and bran.

The elution conditions were as follows: ACQUITY UPLC®BEH C_18_ column (2.1 mm × 50 mm, 1.7 μm); the mobile phase: A was 0.1% formic acid water, and B was acetonitrile; The flow rate was 0.3 ml/min, and the injection volume was 1 μl: column temperature: 30°C; gradient elution: 0→0.6 min, 95% A; 0.6→0.8 min, 95%→89% A; 0.8→2.0 min, 89% A; 2.0→4.0 min, 89%→82% A; 4.0→4.5 min, 82%→81% A; 4.5→7.0 min, 81% A; 7.0→8.5 min, 81%→5% A; 8.5→9.0 min, 5% A; 9.0→9.5 min, 5%→95% A; 9.5→12.0 min, 95% A.

### 2.9 Statistical Analysis

All experimental data were analyzed by GraphPad Prism 8.0.2 (GraphPad InStat Software, San Diego, CA, United States). One-way analysis of variance (one-way ANOVA) or t-test was used to compare each group. All experimental data were expressed by mean ± standard deviation (SD). Meanwhile, the value of *p < 0.05 was statistically significant.

## 3 Results

### 3.1 Effects of Each Fraction in Radix Paeoniae Alba on the Total Number of Monocytes and the Number of Eosinophils and Neutrophils in Bronchoalveolar Lavage Fluid

**FIGURE 2:**
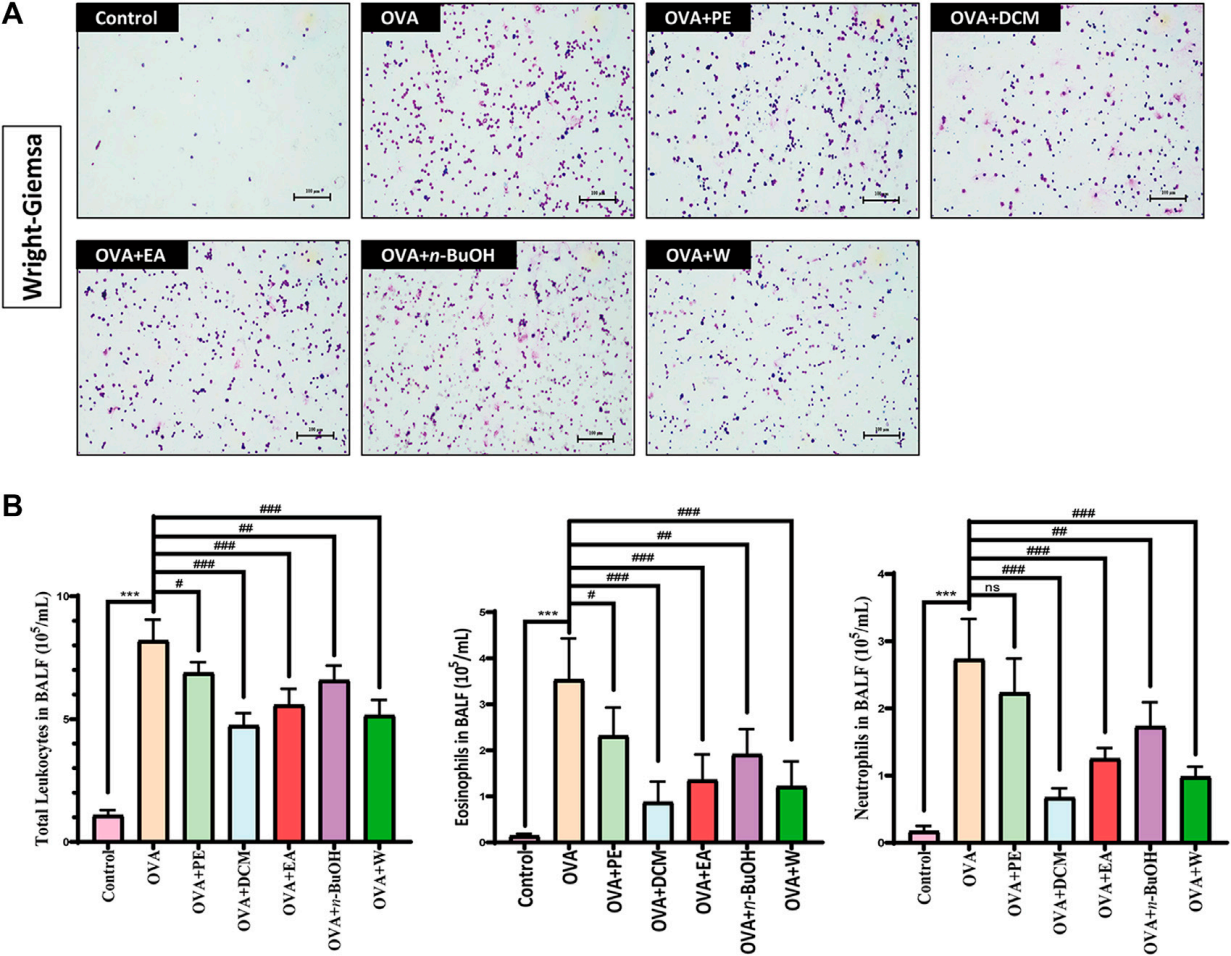
Wright–Giemsa staining and cell count results. **(A)** Results of Wright–Giemsa staining by using a microscope. Scale bar, 100 μm. **(B)** Total number of monocytes, eosinophils, and neutrophils in BALF of each fraction in RPA (*n* = 5). The data were analyzed using one-way ANOVA and are presented as the mean ± SD. *p < 0.05 was statistically significant. *Compared with control, *p < 0.05, **p < 0.01, ***p < 0.001; ^#^compared with model, ^#^ *p* < 0.05, ^##^ *p* < 0.01, ^###^ *p* < 0.001, ns, not significant.

In this section, the effects of each fraction of RPA on the total number of monocytes, the number of eosinophils, and neutrophils in the BALF of OVA-sensitized mice were determined in the experiment. The results of the Wright–Giemsa staining are shown in Figure 2A. The experimental results of cell count are shown in Figure 2B: the BALF cell counts were compared with the control, the BALF total number of monocytes, the number of eosinophils, and neutrophils were significantly increased in the OVA-sensitized allergic asthma model group (*p* < 0.001), and the number of eosinophils indicated that the OVA-sensitized mice in allergic asthma experiment were successful. The mice treated with DCM, EA, and W had significantly lower BALF total number of monocytes, the number of eosinophils, and neutrophils (*p* < 0.001) than the OVA-induced allergic asthma model group, especially DCM. These experimental results indicated that DCM was the most active fraction in RPA to anti-allergic asthma.

### 3.2 Effect of Each Fraction in Radix Paeoniae Alba on Lung Tissue Pathology in OVA-Sensitized Allergic Asthma

**FIGURE 3:**
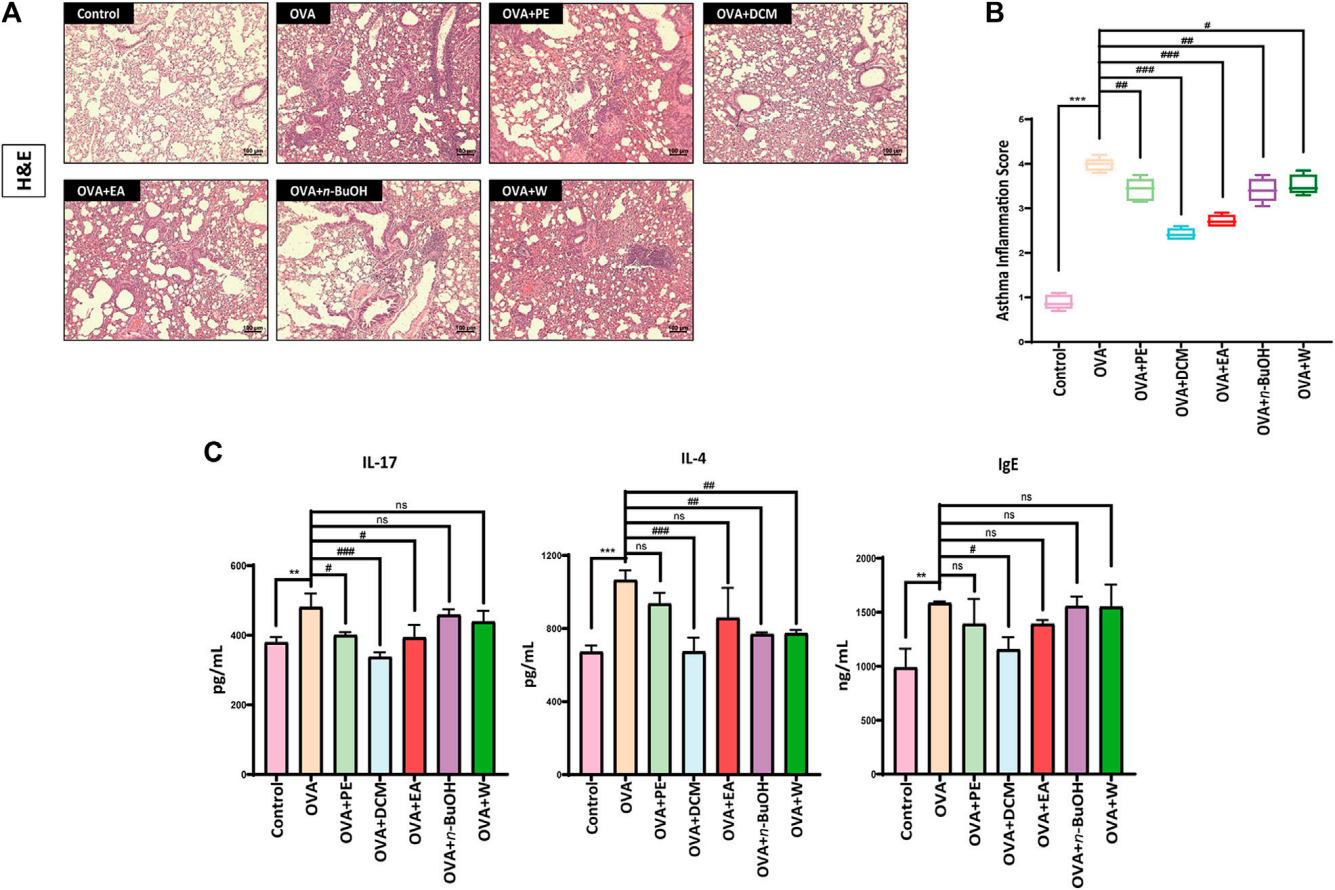
H&E staining, asthma inflammation score and ELISA results. **(A)** Pathological changes of mice lung tissue treated with each fraction from RPA. H&E staining was used to stain lung sections (*n* = 5). Scale bar, 100 μm. **(B)** The results of asthma inflammation score about each fraction from RPA. **(C)** Expression levels of cytokines in the serum (*n* = 3). The data were analyzed by using one-way ANOVA and are presented as mean ± SD. *p < 0.05 was statistically significant. *Compared with control, *p < 0.05, **p < 0.01, ***p < 0.001; ^#^compared with model, ^#^ *p* < 0.05, ^##^ *p* < 0.01, ^###^ *p* < 0.001, ns, not significant.

After H&E staining, the pathological sections of mice were observed by using an optical microscope. The pathological section results of each fraction in RPA are shown in Figure 3A. The results of the pathological sections showed that the alveolar size of the mice in the control was normal, and there was no significant infiltration of inflammatory cells. However, in the OVA-sensitized model group, the bronchus of the lung tissue was infiltrated deeply by inflammatory cells and could be seen that the alveolar area was significantly enlarged, etc. The administration of each fraction from RPA had different degrees of improvement about the inflammation in lung tissue, which included the degree of inflammatory infiltration by inflammatory cells, and the degree of moderation about the pathological structure of lung tissue. The results of the asthma inflammation score are shown in Figure 3B, the scores of the control were about 1 point, and the OVA-sensitized model group was about 4 points. The lower scores the group got, the better improvement it had. The experimental results showed that DCM and EA had a good improvement effect (*p* < 0.001), but DCM was better. The results in this section verified that DCM was the most active fraction in RPA to anti-allergic asthma once again.

### 3.3 Effect of Each Fraction in Radix Paeoniae Alba on the Expression Levels of IL-17, IL-4, and IgE in OVA-Sensitized Allergic Asthma

The occurrence of allergic asthma was related to the mediation of IL-17. In OVA-sensitized mice without any treatment, IL-17 was increased mildly in the serum (*p* < 0.01), whereas in OVA-sensitized mice fed different fractions of each fraction from RPA, IL-17 was differently diminished in the serum, and DCM had a vigorous reducing effect (*p* < 0.001).

IL-4 was produced by CD4 T cells stimulated by antigen or mitogen, which was closely related to the occurrence of allergic asthma. The OVA-sensitized mice had obviously enhanced IL-4 levels in the serum (*p* < 0.001). In OVA-sensitized mice treated with different fractions from RPA, there were varying degrees of the significantly decreased IL-4 levels in the serum, and DCM played the best inhibition effect compared with other treatment groups (*p* < 0.001).

IgE was produced by the plasma cells in the lamina propria of the respiratory and digestive tract mucosa and was one of the immune features that produce allergic asthma. After OVA-sensitized, the IgE levels in the serum were slightly increased compared with the control (*p* < 0.01), whereas in the OVA-sensitized mice gaining each fraction from RPA treatment, to a certain extent, only DCM could reduce IgE levels (*p* < 0.05).

The cytokine expression levels of IL-17, IL-4, and IgE are shown in Figure 3C. These results also proved that DCM was the most active fraction in the treatment of anti-allergic asthma in RPA.

### 3.4 Effects of DCM and FDCM on the Total Number of Monocytes and the Number of Eosinophils and Neutrophils in Bronchoalveolar Lavage Fluid

**FIGURE 4:**
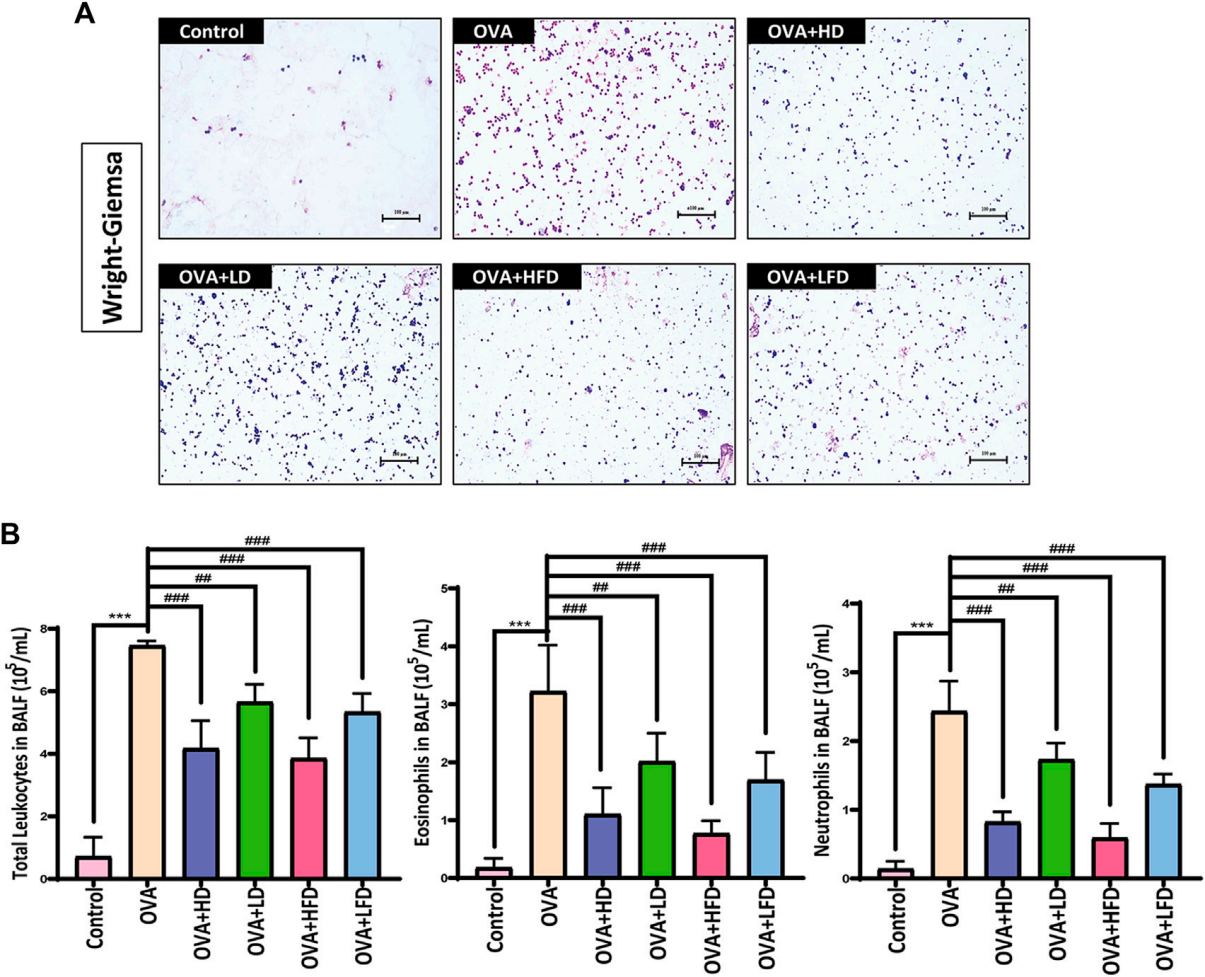
Wright–Giemsa staining and cell count results. **(A)** Results of Wright–Giemsa staining by using a microscope. Scale bar, 100 μm. **(B)** Total number of monocytes, eosinophils, and neutrophils in BALF of high and low doses from DCM and FDCM (*n* = 5). The data were analyzed by using one-way ANOVA and are presented as mean ± SD. *p < 0.05 was statistically significant. *p < 0.05 was statistically significant. *Compared with control, *p < 0.05, **p < 0.01, ***p < 0.001; ^#^compared with model, ^#^ *p* < 0.05, ^##^ *p* < 0.01, ^###^ *p* < 0.001.

In the study of screening the most active fraction from RPA for anti-allergic asthma, the results confirmed that the most active fraction of RPA of anti-allergic asthma was DCM. And in this section, the experiments of the effects of the high and low doses from DCM (HD, LD) and FDCM (HFD, LFD) on the total number of monocytes and the number of eosinophils and neutrophils in BALF of OVA-sensitized mice were determined to explore the enhancement for anti-allergic asthma effects by FDCM. The results of the Wright–Giemsa staining are shown in Figure 4A. The experimental results of cell count are shown in Figure 4B: the BALF cell counts were compared with the control, the BALF total number of monocytes, the number of eosinophils, and neutrophils were sharply increased in the OVA-induced model group (*p* < 0.001), and the number of eosinophils manifested that the OVA-sensitized mice in the allergic asthma experiment were successful. The mice treated with HD, HFD, and LFD had significantly a lower BALF total number of monocytes, the number of eosinophils, and neutrophils (*p* < 0.001) than the OVA-sensitized allergic asthma model group, especially HFD. These experimental results indicated that the dichloromethane fraction from FRPA had a stronger effect on anti-allergic asthma than from RPA.

### 3.5 Effect of DCM and FDCM on Lung Tissue Pathology in OVA-Sensitized Allergic Asthma

**FIGURE 5:**
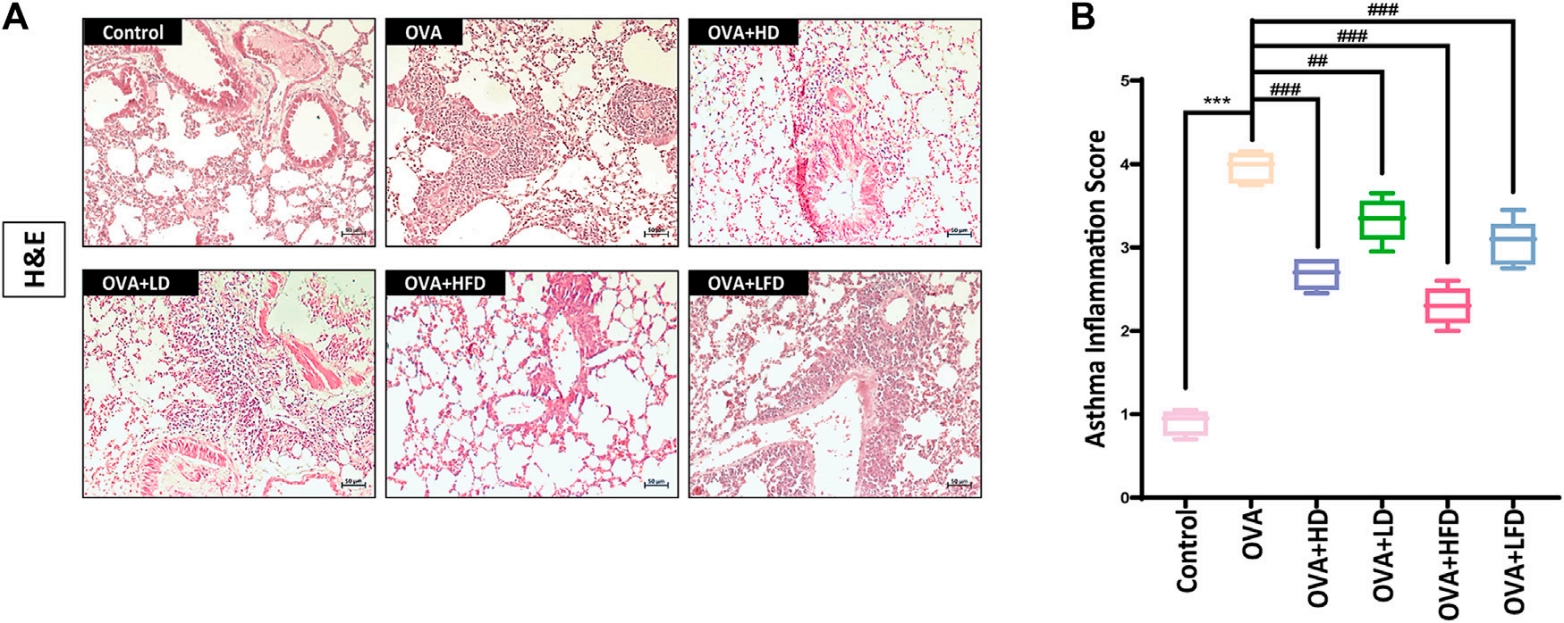
H&E staining and asthma inflammation score. **(A)** Pathological changes of mice lung tissues treated with high and low doses from DCM and FDCM. H&E staining was used to stain lung sections (*n* = 5). Scale bar, 50 μm. **(B)** The results of asthma inflammation score about high and low doses from DCM and FDCM. The data were analyzed by using One-way ANOVA and are presented as mean ± SD. *p < 0.05 was statistically significant. *Compared with control, *p < 0.05, **p < 0.01, ***p < 0.001; ^#^compared with model, ^#^ *p* < 0.05, ^##^ *p* < 0.01, ^###^ *p* < 0.001.

Furthermore, the histopathology of OVA-sensitized mice lungs sections was examined by H&E. The pathological section results are shown in Figure 5A. The results of pathological sections showed that there was no significant infiltration of inflammatory cells in the control, and the mice’s alveolar size was normal. Nevertheless, the bronchus in the lung tissue of the OVA-sensitized model group was infiltrated extremely by the inflammatory cells and could be seen that the alveolar area was markedly enlarged. The administration of each group had different degrees of improvement about the inflammation in lung tissue, which included the degree of moderation about the pathological structure of lung tissues and the degree of inflammatory infiltration by inflammatory cells. The results of the asthma inflammation score are shown in Figure 5B, the scores of the control were about 1 point, and the OVA-sensitized model group was about 4 points. The higher scores the group got, the worse improvement it was. The experimental results showed that HD, HFD, and LFD had a good improvement effect (*p* < 0.001), but HFD was best. In general, the experimental results confirmed that the dichloromethane fraction from FRPA had a better effect on anti-allergic asthma than from RPA once again.

### 3.6 Effect of DCM and FDCM on the Expression Levels of Inflammatory Factors IL-1β and IL-6 in OVA-Sensitized Allergic Asthma

**FIGURE 6:**
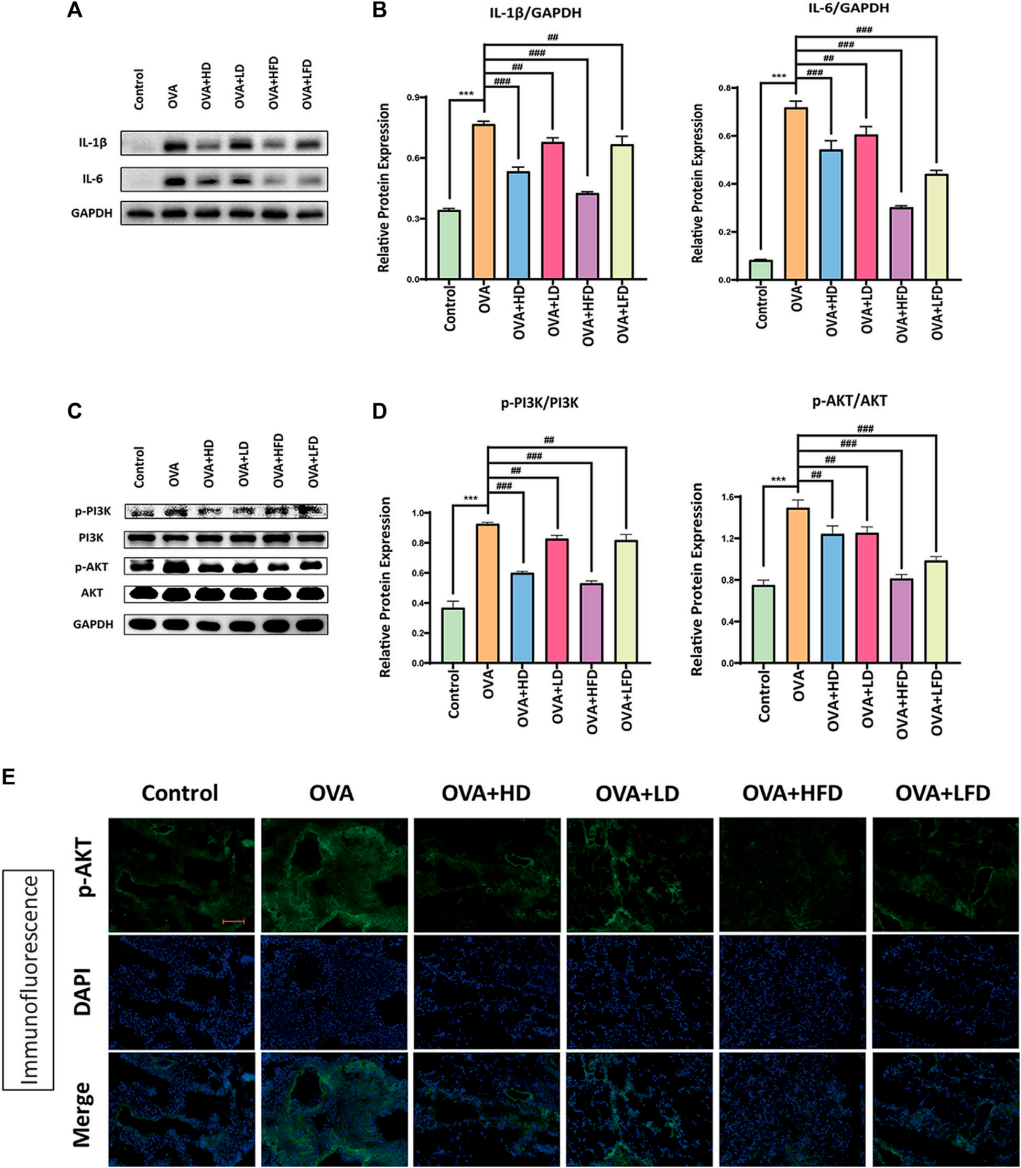
Western blotting and Immunofluorescence staining results. **(A)** Representative Western blotting analysis for expressions of IL-1β and IL-6. Densitometric values were normalized against GAPDH. **(B)** Relative protein expression of IL-1β and IL-6 in OVA-sensitized mice with the treatment of high and low doses of DCM and FDCM. **(C)** Representative Western blotting analysis for expressions of p-PI3K, PI3K, p-AKT and AKT. Densitometric values were normalized against GAPDH. **(D)** Relative protein expression of p-PI3K and p-AKT OVA-sensitized mice with the treatment of high and low doses of DCM and FDCM. **(E)** Immunofluorescence staining of p-AKT. Scale bar, 200 µm. Protein bands were analyzed by ImageJ (*n* = 3). GAPDH was used as internal controls. The data were analyzed by using one-way ANOVA and presented as mean ± SD. *p < 0.05 was statistically significant. *Compared with control, *p < 0.05, **p < 0.01, ***p < 0.001; ^#^Compared with model, ^#^ *p* < 0.05, ^##^ *p* < 0.01, ^###^ *p* < 0.001.

IL-1β and IL-6 were two inflammatory factors related to allergic asthma. For the sake of exploring the regulation of inflammatory factors of FDCM in OVA-sensitized allergic asthma, the expression of IL-1β and IL-6 was detected by Western blotting. The primary antibody of IL-1β, IL-6, and GAPDH was used to detect the protein expression levels. The protein expressions of associated bands are shown in Figure 6A. The results illustrated that OVA could enhance the levels of IL-1β and IL-6, while the effects could be diminished by treatment about high and low doses of DCM and FDCM. In short, HFD could decrease the protein expression of IL-1β and IL-6 when compared with the OVA-sensitized model group (*p* < 0.001). The correlative bands analysis results were presented in Figure 6B. These findings asserted that the dichloromethane fraction from FRPA could enhance the effect of anti-allergic asthma under OVA-sensitized by regulating inflammatory factors IL-β and IL-6.

### 3.7 Effect of DCM and FDCM on the Activation of PI3K/AKT Signaling Pathway in OVA-Sensitized Allergic Asthma

The PI3K/AKT signaling pathway was one of the common signaling pathways in immunosuppression and inflammatory diseases. Also in order to explore the signaling pathway of FDCM in OVA-sensitized allergic asthma, the expression of PI3K and AKT was detected by Western blotting. The primary antibodies of p-PI3K, PI3K, p-AKT, AKT, and GAPDH were used to detect the protein expression levels. The protein expression results of the relevant bands are shown in Figure 6C. The results indicated that OVA could enhance the levels of p-PI3K and p-AKT, while these effects could be decreased by treatment of high and low doses of DCM and FDCM. In brief, the HFD could prevent the phosphorylation of PI3K and AKT when compared with the OVA-sensitized model group (*p* < 0.001). The band analysis results are presented in Figure 6D. These findings suggested that the dichloromethane fraction from FRPA could enhance the effect of anti-allergic asthma under OVA-sensitized by targeting the PI3K/AKT signaling pathway.

### 3.8 Effect of DCM and FDCM on the Expression Level of p-AKT in Lung Tissues by Immunofluorescence Analysis in OVA-Sensitized Allergic Asthma

Immunofluorescence staining of p-AKT further verified the results of the Western blotting, and the immunofluorescence results are presented in Figure 6E. The immunofluorescence analysis of frozen sections of FRPA lung tissues with p-AKT showed that HD and HFD could inhibit the expression of AKT phosphorylation in the lung tissue of OVA-induced allergic asthma mice, but HFD had a better inhibition effect. The experimental results also proved that the FDCM had a significant anti-allergic effect on asthma, and the effect was better than that of DCM.

### 3.9 Changed Contents of Main Compounds in FDCM Compared With DCM

**FIGURE 7:**
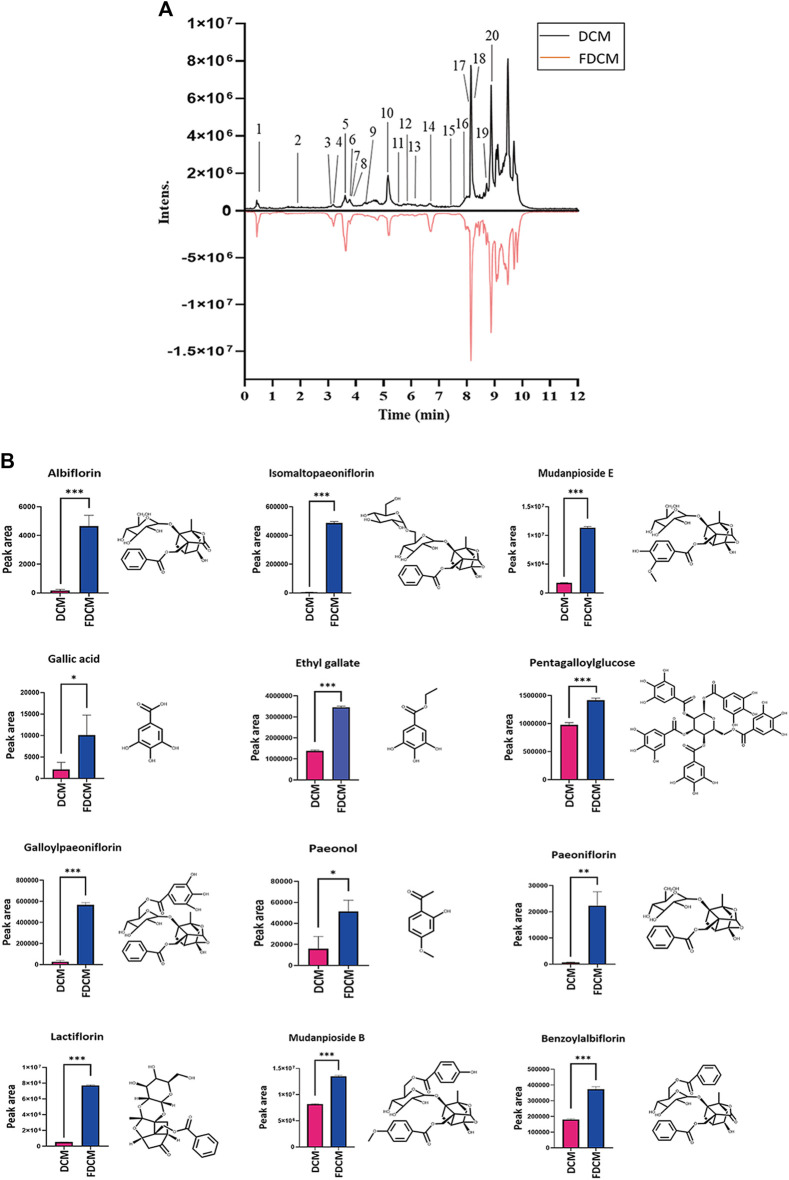
UPLC analysis results and bar chart of compounds contents. **(A)** Chromatograms of UPLC on DCM and FDCM. **(B)** Bar charts about the significantly increased contents of some compounds in FDCM compared with DCM, which included compound structures (*n* = 3). The data were analyzed using a T-test and presented as mean ± SD. *p < 0.05 was statistically significant. Compared with DCM, *p < 0.05, **p < 0.01, ***p < 0.001.

The pharmacological experimental results confirmed that FDCM had stronger pharmacological effects on anti-allergic asthma than DCM. In this section, the experiment analyzed further to explore the main components in DCM and FDCM and the changed contents of FDCM after stir-frying by honey and bran. Therefore, UPLC/Q-TOF-MS was used to analyze the samples of FDCM and DCM. The chromatograms of UPLC are shown in Figure 7A.

Through UPLC/MS software analysis, the experimental results confirmed that the contents of many components in FDCM were changed after stir-frying by honey and bran. And the compounds were identified by the pharmacopoeia standards, the reported compounds, and mass spectrometry. The results are summarized in Table 1. Subsequently, the research performed significant difference analysis on the average values of their peak area by using a t-test, and at last, the experimental results manifested that the contents of many compounds in FDCM were significantly increased with a statistical difference after stir-frying with honey and bran. The bar charts of comparisons of the contents of some compounds are presented in Figure 7B.

**TABLE 1:** Changed content of main compounds in FDCM compared with DCM.

No.	Name	Formula	t_R_ (min)	Ion mode	Mass (m/z)	Typical fragment ions (m/z)	Peak area		References
							RPA	FRPA	
1	Sucrose	C_12_H_22_O_11_	0.45	[M−H]^-^	341.1078	119, 89, 71, 59	309,725	1,732,254	Ren et al. (2019)
2	Oxypaeoniflorin	C_23_H_28_O_12_	1.99	[M+CH_2_O_2_−H]^-^	541.1553	495, 465, 333, 137	28	218	Ren et al. (2019)
3	Albiflorin	C_23_H_28_O_11_	3.18	[M−H]^-^	479.1552	319, 197, 159, 129	169	4,654	Zheng et al. (2015)
4	Isomaltopaeoniflorin	C_29_H_38_O_16_	3.20	[M+CH_2_O_2_−H]^-^	687.2143	593, 525, 323, 121	5,121	487,242	Tan et al. (2017)
5	Mudanpioside E	C_24_H_30_O_13_	3.63	[M−H]^−^	525.1605	449, 327, 165, 121	1,757,345	11,362,228	Zheng et al. (2015)
6	Gallic acid	C_7_H_6_O_5_	3.78	[M−H]^−^	169.0132	171, 153, 127, 109	2072	10,094	Zheng et al. (2015)
7	Ethyl gallate	C_9_H_10_O_5_	3.79	[M−H]^−^	197.0444	197, 169, 125, 124	1,377,324	3,459,845	Yang et al. (2021)
8	Pyrogallol	C_6_H_6_O_3_	3.81	[M−H]^−^	125.0240	125, 108, 95, 81	611	6,112	Ren et al. (2019)
9	Ellagic acid	C_14_H_6_O_8_	4.37	[M−H]^−^	300.9988	300, 257, 229, 201	30,490	27,915	Ren et al. (2019)
10	Pentagalloylglucose	C_41_H_32_O_26_	5.18	[M−H]^−^	939.1095	939, 769, 469, 451	975,683	1,414,974	Zheng et al. (2015)
11	Galloylpaeoniflorin	C_30_H_32_O_15_	5.49	[M−H]^−^	631.1661	631, 601, 313, 169	26,455	564,717	Zheng et al. (2015)
12	Paeonol	C_9_H_10_O_3_	5.86	[M−H]^−^	211.0601	211, 196, 166, 123	15,827	51,269	Zheng et al. (2015)
13	Paeoniflorin	C_23_H_28_O_11_	6.21	[M−H]^−^	479.1554	525, 509, 479, 121	625	22,346	Zheng et al. (2015)
14	Lactiflorin	C_23_H_26_O_10_	6.68	[M + CH_2_O_2_−H]^−^	507.1501	507, 339, 177, 121	525,724	7,703,073	Zheng et al. (2015)
15	Ethyl 3,4-dihydroxy-5-[(3,4,5-trihydroxybenzoyl)oxy]benzoate	C_16_H_14_O_9_	7.60	[M−H]^−^	349.0577	197, 169, 124	51,757	17,656	Wu L. F. et al. (2020)
16	Pinen-10-yl vicianoside	C_21_H_34_O_10_	7.98	[M + CH_2_O_2_−H]^−^	491.2123	445, 293, 233, 191	29,794	1,355,835	Tan et al. (2017)
17	Mudanpioside B	C_31_H_34_O_14_	8.15	[M−H]^−^	629.1861	553, 431, 165, 121	8,176,472	13,453,885	Zheng et al. (2015)
18	Benzoylalbiflorin	C_30_H_32_O_12_	8.17	[M−H]^−^	583.1816	583, 551, 431, 121	178,756	372,997	Zheng et al. (2015)
19	30-Norhederagenin	C_29_H_44_O_4_	8.72	[M−H]^−^	455.3157	455, 409	319,396	563,943	Ren et al. (2019)
20	Hederagenin	C_30_H_48_O_4_	8.90	[M−H]^−^	471.3465	471, 435, 407, 325	531,821	537,677	Zheng et al. (2015)

## 4 Discussion

Over the past decades, allergic asthma and airway inflammatory disease had exerted great impacts on human and animal health worldwide. At present, most medical researchers believed that the main factor causing asthma was the imbalance of Th1/Th2 and Th17/Treg. At present, most medical researchers believed that the main factor causing asthma is the imbalance of Th1/Th2 and Th17/Treg, and the regulation of PI3K/AKT and other signaling pathways can effectively control the progression of asthma. PI3K was a heterodimer protein consisting of a catalytic subunit and a regulatory subunit. Under basic conditions, regulatory proteins would bind, stabilize, and inhibit catalytic subunits. During cell activation, the regulatory proteins would contact the catalytic subunits with a lipid substrate on the membrane. PI3K would be activated by Ras, RTKs, and GPCRs, and after activation, the activated PI3K was able to phosphorylate PIP2 to produce PIP3, causing the recruitment of PDK1 and AKT to the plasma membrane. Then, PDK1 and mTORC2 would phosphorylate AKT. Finally, the activated AKT was able to phosphorylate plenty of downstream effectors, which regulated the cell growth, cell survival, angiogenesis, and migration, or invasion. In the previous research, Wu et al. found through experiments that alpinetin had good anti-inflammatory activity against allergic asthma by regulating PI3K/AKT/NF-κB and HO-1 signaling pathways (Wu D. et al., 2020). Ma et al. mentioned in the study that allergic asthma would activate the mTOR signaling pathway, which led to the upregulation of p-PI3K, p-AKT, and p-mTOR. The general pathway can be simply described as PI3K was activated by external stimulation and inflammation, and after activation, p-PI3K was formed, which would phosphorylate AKT into p-AKT and finally activate mTOR, translate protein, and grow cells (Ma et al., 2021). Chen Xi et al., through experiments, found that surfactant protein A can inhibit allergic reactions of asthma mice induced by OVA by regulating the activity of JAK/STAT (Chen et al., 2021). *Vitex negundo* Linn. extracts can regulate the AMPK/PI3K/AKT/p38-NF-κB and TGF-β/Smad/Bcl2/caspase/LC3 signaling pathways to produce cascade reaction and activate macrophages, thus alleviating allergic asthma and lung infection induced by OVA (Tirpude et al., 2021).

In this study, the most effective fraction of RPA that exerts the anti-allergic asthma effect was evaluated, and after processing honey and bran, the pharmacological effect, pharmacological mechanisms of action, and material basis of increasing the anti-allergic asthma effect of the effective active fraction in FRPA were further explored. First, RPA was extracted, and the drug components of each fraction were obtained by using different polar extracts. Subsequently, the research evaluated the most active fraction for anti-allergic asthma by OVA sensitization of female BALB/c mice to model and administer drugs to each fraction of RPA. According to the experimental results of histopathological analysis, the lung tissues of OVA-induced BALB/c mice showed obvious inflammatory infiltration, and the alveolar structure was also significantly damaged. Based on the results of H&E staining, DCM, the dichloromethane fraction of RPA, could significantly reduce the degree of inflammatory infiltration of lung tissue. On the other hand, in the process of airway inflammation, there were many inflammatory cells involved, including eosinophils and neutrophils. Eosinophils were the main participants in the process of allergic asthma. Judging from the cell count after Wright–Giemsa staining of BALF, DCM can prevent the alveolar area from being infiltrated by inflammatory cells, especially eosinophils, to some extent. The experimental results were similar to those of pulmonary histopathological analysis, indicating that DCM had a good anti-allergic effect on asthma. T cells differentiated into Th1, Th2, and Th17 after antigen exposure and receptor stimulation. IL-17 produced by Th17 cells can stimulate bronchial epithelial cells in allergic asthma and had a certain effect on the production of cytokines and chemokines. IL-4 produced by Th2 cells played a central role in the development of asthma, in that it increased the expression level of inflammatory cytokines in the lung and ultimately stimulated the production of IgE. Inspired by the fact that IgE can mediate an allergic immune response, it was the main regulator of Th2 cytokines that controlled the progression of asthma (Aghajani et al., 2019; Wu D. et al., 2020). The results of ELISA showed that DCM could effectively reduce the expression levels of IgE, IL-17, and IL-4 in serum. The aforementioned experimental results showed that DCM was the most active effective fraction for anti-allergic asthma in RPA.

Afterward, the dichloromethane fraction from FRPA was obtained according to the RPA-related extraction and the separation process to evaluate the enhancement of pharmacological effect after the processing with honey and bran. From the results of H&E staining, the high dose of the dichloromethane fraction from FRPA did exert a stronger pharmacological effect than the high dose of the dichloromethane fraction from RPA, and the pathological sections of the lung tissue showed the best inhibition degree of inflammatory infiltration compared with other groups. The cell count results of BALF also showed that HFD could reduce the number of eosinophils and neutrophils more effectively than HD. In addition, the research further studied the inflammatory factors and signaling pathways of FDCM against OVA-induced allergic asthma. Th2 cells secreted IL-6 to prevent allergic diseases (Jian et al., 2013). IL-1β can stimulate Th2 cells and increase the eosinophils in the lung inflammation site, thus mediating the development process of allergic asthma (Sobkowiak et al., 2017). In Western blotting analysis, it was found that HFD significantly reduced the contents of IL-1β and IL-6, and HFD was better than HD. Furthermore, pathologies such as inflammatory cell infiltration can be suppressed by inhibiting the activation of PI3K and AKT in mouse models of allergic asthma (Wu D. et al., 2020). FDCM was found to be capable of inhibiting the expression levels of p-PI3K and p-AKT. At the same time, according to the analysis results of immunofluorescence, it was found that HFD could effectively inhibit the expression of AKT phosphorylation in the lung tissue. These experimental results indicated that FDCM had potential pharmacological mechanisms of action to exert anti-allergic asthma by targeting the PI3K/AKT signaling pathway.

Furthermore, the better pharmacological action of the dichloromethane fraction of FRPA was due to the increased contents of its main components after processing with honey and bran, which might be because the carbohydrate-rich honey played a protective role in the structure of the main chemical components of the traditional Chinese medicine during the high-temperature processing (Ao et al., 2020). The main components of the dichloromethane fraction from FRPA were generally paeoniflorin and its structure analogous compounds. The experimental results indicated that the components of paeoniflorin and the structure analogous compounds of paeoniflorin like galloylpaeoniflorin were significantly increased in the dichloromethane fraction from FRPA after processing. Paeoniflorin can reduce the expression levels of IL-5, IL-13, IL-17, and eotaxin in the OVA-induced allergic asthma mice model, which may be concerned with the blocking of the MAPK pathway activation (Zhou et al., 2020). Galloylpaeoniflorin can alleviate PM2.5-induced vascular endothelial cell injury on inflammatory responses, which can be achieved by activating the Nrf2 signaling pathway (Bi et al., 2020). In addition, there were also some compounds with other structures that showed a significant increase, such as ethyl gallate and pentagalloylglucose. To a certain extent, ethyl gallate and pentagalloylglucose can protect the LPS-induced acute lung injury by inhibiting the number of polymorphonuclear leukocytes in BALF, reducing the wet/dry ratio and protein concentration of the lung, and improving the permeability of the lung (Zhang et al., 2018). The research group is currently conducting research on their pharmacological activities and other aspects.

Coronavirus disease 2019 (COVID-19) is a global infectious disease, caused by severe acute respiratory syndrome coronavirus 2 (SARS-CoV-2), making it one of the severe public health issues in recent decades, which had put a heavy toll on public health, lives, and the world economy. Hundreds of molecular tests and immunoassays were rapidly developed (Vandenberg et al., 2021), and several agents were being evaluated (Gavriatopoulou et al., 2021). However, most of the current guidelines did not specifically advise on how to treat pneumonia and acute respiratory distress syndrome, which were the major complications of COVID-19, such as cough and inflammation (Anka et al., 2021). RPA was a well-known natural medicine in China already widely available for the management of respiratory conditions, mainly regarding the symptoms (inflammation and immune function). Our work suggests that RPA had evidence to the discussion about its potential use as adjuvants in the treatment of early/mild common flu in otherwise healthy adults within the context of COVID-19.

## Data Availability

The original contributions presented in the study are included in the article/Supplementary Material, further inquiries can be directed to the corresponding author.

## Abbreviations

BALF, bronchoalveolar lavage fluid; BCA, bicinchoninic acid; DCM, dichloromethane fraction of RPA; EA, ethyl acetate fraction of RPA; ECL, enhanced chemiluminescence; FDCM, dichloromethane fraction of FRPA; FEA, ethyl acetate fraction of FRPA; Fn-BuOH, n-butanol fraction of FRPA; FPE, petroleum ether fraction of FRPA; FRPA, stir-frying Radix Paeoniae Alba with honey and bran; FW, water fraction of FRPA; H&E, hematoxylin and eosin staining; HD, DCM high-dose group; HFD, FDCM high-dose group; LD, DCM low-dose group; LFD, FDCM low-dose group; *n*-BuOH, *n*-butanol fraction of RPA; one-way ANOVA, one-way analysis of variance; OVA, ovalbumin; PBS, phosphate-buffered saline; PBST, phosphate-buffered saline with Tween-20; PE, petroleum ether fraction of RPA; PVDF, polyvinylidene fluoride; RA, rheumatoid arthritis; RPA, Radix Paeoniae Alba; SD, standard deviation; SDS-PAGE, sodium dodecyl sulfate–polyacrylamide gel electrophoresis; W, water fraction of RPA.
